# CoviSwin: A Deep Vision Transformer for Automatic Segmentation of COVID-19 CT Scans

**DOI:** 10.3390/bioengineering12111227

**Published:** 2025-11-10

**Authors:** Alhanouf Alsenan, Belgacem Ben Youssef, Haikel S. Alhichri

**Affiliations:** 1 Department of Computer Engineering, King Saud University, P.O. Box 51178, Riyadh 11543, Saudi Arabia; alhanouf.alsenan@gmail.com; 2 Department of Computer Science and Technology, Champlain College, 900 Riverside Dr., Saint-Lambert, QC J4P 3P2, Canada; haikel.alisr@gmail.com

**Keywords:** vision transformer, swin transformer, swin transformer version 2, deep learning, COVID-19, image segmentation, dice similarity coefficient, sensitivity, specificity

## Abstract

Precise segmentation of COVID-19 lesions in chest Computed Tomography (CT) scans can directly impact patient care, yet existing methods struggle, when undertaking this task, with the heterogeneous appearance of ground-glass opacities, consolidations, and the availability of limited labeled data. We propose herein CoviSwin, a Transformer-based U-shaped encoder–decoder network that combines the Large model of Swin Transformer Version 2 with attention and residual connections to capture both global context and fine details. A two-phase training strategy is applied whereby in the first phase the encoder is initially frozen while training the decoder on the public SemiSeg dataset, then in the second phase, the encoder is partially unfrozen while the whole model is trained on the publicly available MedSeg dataset. The model achieves a ten-run mean sensitivity value of 0.790 ± 0.012, an average Dice Similarity Coefficient (DSC) score of 0.781 ± 0.0068, and an average specificity of 0.962 ± 0.0049, outperforming the sensitivity results obtained by recent models such as NextSeg of 2024 and GFNet of 2022 by 8.07% and 7.48%, respectively. These findings demonstrate the potential of CoviSwin as an effective model for clinical COVID-19 lesion segmentation.

## 1. Introduction

Medical image segmentation is a cornerstone of modern medical imaging, enabling precise identification of organs, tissues, and pathological anomalies in Magnetic Resonance Imaging (MRI), Computed Tomography (CT) and UltraSound (US) scans. It is an essential tool in diagnosing a disease, treatment, and even confirming the progression of the disease in patients. At the end of 2019, a novel coronavirus was identified as the cause of severe respiratory illness in humans. Although related to the coronavirus responsible for the 2003 Severe Acute Respiratory Syndrome (SARS) outbreak, this new virus was designated Severe Acute Respiratory Syndrome CoronaVirus 2 (SARS-CoV-2). It is the causative agent of coronavirus disease 2019 (COVID-19). On 11 March 2020, the World Health Organization (WHO) declared COVID-19 a global pandemic. Since then, the disease has affected more than 160 million people worldwide while contributing to the death of seven million people [1].

The pandemic underscored the need for advanced imaging techniques to assist in diagnosis, monitoring, and treatment planning for affected patients [2]. Chest X-Rays (CXR) and CT scans became essential for evaluating lung infections and complications. However, manual interpretation is time-consuming and prone to inter-observer variability, spurring a surge of automated segmentation methods to accurately delineate infected lung regions. Automated medical image segmentation not only quantifies disease burden and severity but also supports clinicians in making timely and well-informed decisions.

Traditionally, Convolutional Neural Networks (CNNs) have been the backbone of medical image analysis due to their ability to extract spatial features effectively. Yet, CNNs are limited by their local receptive fields, which can restrict their capacity to model global contexts, a critical aspect in understanding the spread and structure of COVID-19 related lung infections. To address these limitations, researchers began exploring the use of Transformer-based models, particularly Vision Transformers (ViTs), which were originally designed for image classification tasks in the field of computer vision, inspired by the early success of Transformers in natural language processing [3]. ViTs introduce a self-attention mechanism that allows the model to capture long-range dependencies and global contextual relationships across the entire image. In the context of medical segmentation, particularly for COVID-19, this capability facilitates a more holistic understanding of infection patterns across different regions of the lungs. Unlike CNNs, which process data in a strictly localized manner, ViTs can weigh the importance of every pixel relative to other pixels in the image, enabling precise delineation of complex and diffuse patterns commonly observed in COVID-19 infections.

ViTs represent a paradigm shift in medical image segmentation by leveraging attention mechanisms to effectively model both local features and global contextual relationships within an image [4]. Recent studies and implementations have shown that ViT-based segmentation models outperform traditional CNN-based methods in terms of accuracy and robustness. Hybrid models that combine CNN-based encoders with ViT decoders, or entirely Transformer-based architectures like TransUNet and UNETR, have further pushed the boundaries in this area. The integration of these models into clinical workflows holds promise for real-time, AI-assisted diagnosis that could significantly ease the burden on healthcare systems, particularly during global health crises.

Continuing with our previous work on the segmentation of medical images [5], we employ in this research a Transformer model based on a UNet-style encoder–decoder architecture for the segmentation of COVID-19 lesions. Motivated by the success of the Swin Transformer [6] and SwinUNet [7] models, we propose herein CoviSwin, a fully Transformer-based U-shaped segmentation framework tailored for 2D medical images. Building on the enhancements provided by Swin Transformer Version 2 (V2) and SwinUNet models, CoviSwin incorporates a hierarchical attention mechanism, shifted windows, and residual connections to capture both fine-grained spatial details and long-range dependencies. The model is designed to handle the heterogeneous appearance of COVID-19 lesions, ensuring accurate delineation across diverse patient populations and imaging conditions. Furthermore, we adopt a two-phase training strategy to enhance both stability and efficiency. In the first phase, the encoder remains frozen while the decoder is trained on a large pseudo-labeled dataset. This step shifts the decoder’s initial random weights closer to optimal regions of the parameter space. In the second phase, we partially unfreeze the encoder and fine-tune the entire model on the target dataset, enabling more effective transfer and faster convergence to a well-optimized set of weights. In addition to the large-scale model, we develop a lightweight variant suitable for deployment in resource-constrained settings, such as mobile health applications or low-power diagnostic devices. Therefore, the main contributions of this work are summarized as follows:

1. Transformer-based model utilizing a Swin variant deep-learning model: We design a segmentation framework that employs the Swin Transformer V2 Large, heretofore denoted by SwinV2-L with approximately 197 million parameters, as the encoder component. The large size, hierarchical attention structure, and shifted window mechanism of SwinV2 enable multi-scale feature extraction, improving the model’s ability to capture both fine-grained and global contextual information within medical images.
2. Decoder with attention-gated refinement and deep supervision: The decoder incorporates three key enhancements: (i) attention gates within the upsampling/deconvolution blocks to selectively emphasize task-relevant features, (ii) a residual connection in the final decoder block to stabilize gradient flow and preserve fine structural details, and (iii) an auxiliary output head (deep supervision) attached at an intermediate decoder stage. The residual path provides identity “highways” that mitigate vanishing gradients in deep U-shaped encoder–decoder and Transformer blocks, while the auxiliary head shortens the backpropagation path and supplies stronger supervisory signals to earlier layers. Together, these components may enhance feature refinement, improve training stability and convergence, as well as potentially yield more accurate segmentation masks.
3. Two-phase training mechanism: We employ a two-phase training strategy designed to improve both optimization stability and training efficiency. During the first phase, the encoder is kept frozen while the model is trained on a large pseudo-labeled dataset [8]. This initialization step serves to steer the decoder’s parameters away from random initialization and towards more promising regions of the solution space. In the second phase, we selectively unfreeze parts of the encoder and jointly fine-tune the entire model on the target dataset. This progression enables more effective transfer of knowledge from the pseudo-label pretraining stage, accelerates convergence, and could yield a more robust set of optimized parameters.

We also employ, alongside the proposed model, a composite loss function for accurate segmentation, where a tailored loss function combining Dice Loss, Intersection over Union (IoU) Loss, and Binary Cross-Entropy (BCE) Loss functions to optimize segmentation performance. This combined loss formulation addresses challenges such as class imbalance and fuzzy boundaries, commonly observed in medical image datasets, and promotes accurate region-level and boundary-aware segmentation in specific COVID-19 lesions.

The rest of the paper is organized as follows: Section 2 discusses some of the recent work related to this research. Then, in Section 3, we outline the research methodology, including details on the employed datasets, model description, and the adopted experimental setup. Section 4 presents and analyzes the results. Finally, we summarize in Section 5 our conclusions and discuss potential directions for future work.

## 2. Related Work

Convolutional neural networks (CNNs) have long been the dominant approach in the area of medical image segmentation, with several prominent architectures proposed [9]. One of the recent works in this field is the research presented in [10], where the authors introduced the Collaborative Generalist and Specialists (CGS) framework to improve multi-target semi-supervised medical image segmentation. The method uses a generalist network for overall segmentation and specialist networks for each target, with cross-consistency losses and an inter-head error detection module to improve pseudo-label quality. Tests on ACDC, SegTHOR, and Synapse datasets show that CGS achieves better segmentation than previous methods, even when only a small amount of labeled data is available. Additionally, the work in [11] proposed MTAN (Mean Teacher Attention N-Net), a semi-supervised model for kidney and tumor segmentation in CT images. The model uses a teacher-student framework, where the teacher generates pseudo-labels to guide the student, enabling effective use of both labeled and unlabeled data. Evaluations on KiTS19 and KiTS21 datasets showed high Dice scores for kidneys, tumors, masses, and cysts, demonstrating MTAN’s accuracy and robustness even with limited labeled data. These models extract hierarchical features from images to identify structures within medical data.

Despite their effectiveness in image segmentation, CNNs struggle to capture long-range dependencies, which are crucial for accurately segmenting complex or overlapping anatomical regions. In contrast, ViTs divide an image into patches and process them as sequences of tokens, enabling the modeling of long-distance relationships more effectively [3,12]. Rather than comparing each patch with all others, ViTs treat patches as ordered sequences, thereby enhancing spatial feature representation. Nevertheless, standard ViTs are computationally expensive, and much of the recent research has focused on improving their segmentation accuracy while balancing computational cost, making them suitable for a broad range of medical imaging applications.

Research described in [13] states that to overcome these drawbacks, a new class of models called Hybrid Vision Transformers (HVTs) was developed, where the CNN-like hierarchical feature extraction process combined with transformer-style self-attention were combined. The authors reported that these models not only incorporated CNNs’ abilities for local feature extraction but also integrated ViT’s global dependency modeling [13,14]. While vanilla ViT performs global self-attention over all the patches, Swin Transformer segments an image and progressively down-samples while maintaining the semantic meaning of the patches. The significant new elements introduced in this work are the notion of the shifted window attention mechanisms, where the input image is segmented into disjoint windows, and attention is computed separately within each of these windows, as in [6,15]. The model permits cross-window connections by sliding windows, which helps it capture the fine-grained local structures and long-range dependency while reducing computational expenses. This shifted window approach also optimizes memory consumption significantly, making it well-suited to segment large medical images with high resolutions. Among the successful hybrid architectures, the Swin Transformer introduced hierarchical feature representation and a change in the window-attention mechanism to enhance segmentation performance.

In the pursuit of better feature learning while maintaining manageable computational complexity, advanced modifications have been made to the shifted window-based attention mechanisms, as discussed in [16,17]. These enhancements contribute to better perceptual quality and fidelity in image reconstruction tasks. In this context, the two measures of Peak Signal-to-Noise Ratio (PSNR) and Structural Similarity Index (SSIM) are commonly used evaluation metrics, where higher values of both indicate better reconstruction performance. Building on this line of research, recent work has extended transformer-based strategies from reconstruction to segmentation, demonstrating their adaptability across medical imaging tasks. In this regard, the work in [18] investigates Vision Transformer-based architectures, including ARSeg, MedT, TransUNet, TransM, and UNeXt, for the task of lung segmentation in CXR images. Accurate lung segmentation is an essential step in supporting COVID-19 diagnosis and monitoring; however, conventional convolutional neural networks often struggle to capture the global contextual dependencies required for this task. By integrating ViT modules, the proposed models effectively leverage long-range feature interactions, enabling improved delineation of lung regions. The study demonstrates that these transformer-enhanced approaches outperform standard CNN-based baselines, underscoring the potential of ViTs in advancing medical image analysis, particularly in pandemic-related applications.

Unlike ViTs, where self-attention computations scale with the image size, the Swin Transformer restricts attention to localized windows, thereby reducing computational cost without compromising segmentation accuracy. An additional advantage of the Swin Transformer lies in its hierarchical feature extraction mechanism, which enables the effective capture of fine details across multiple scales. By progressively reducing the spatial resolution of feature maps through patch merging, the Swin Transformer learns multi-scale representations while retaining both low-level details and high-level semantic context. This property makes it particularly well-suited for tasks such as image denoising and segmentation, as demonstrated in SUNet [19], where a Swin Transformer-based UNet architecture enhances feature extraction and improves image restoration performance.

This multi-scale learning capability is instrumental in medical image segmentation. Swin Transformer has demonstrated superior performance across multiple medical image segmentation benchmarks [16]. When implemented on the BraTS dataset, higher Dice Similarity Coefficient (DSC) scores have been obtained by comparing to previous CNN and Transformer models, thus demonstrating that the model can segment the brain tumors effectively [20]. Likewise, on the Abdominal Multi-Organ Segmentation (AMOS) dataset, Swin Transformer successfully detects multiple scales of organ structures, resulting in better segmentation [21]. The model also performed well in the Kidney Tumor Segmentation (KiTS) Challenge dataset since it can adapt to the feature representation of the tumor and other structures within the kidney segmentation [21].

Another enhancement of Swin Transformer compared to other models is that it sets segmentation references more accurately. The model performs better by retaining the long-distance dependencies between segments, providing a more accurate and semantically coherent segmentation outcome [22]. In addition, the proposed method has a lower computational complexity than standard ViTs, which makes it more realistic for adoption in clinical applications, especially in scenarios where computational resources are limited. Moreover, hierarchical representation learning can be applied to various modalities in the medical imaging field, improving the performance of models involved in the undertaken task. However, the Swin Transformer is not without challenges. One of its primary limitations is its reliance on large annotated datasets. While CNNs are inherently effective at capturing local spatial relationships, Transformers typically require large-scale labeled data to perform well [12]. This presents a significant obstacle in the medical imaging domain, where annotated datasets are scarce and costly to obtain. As a result, approaches such as data augmentation or self-supervised learning may not be sufficient to overcome this limitation. Another concern is the complexity of the fine-tuning process. The Swin Transformer requires careful hyperparameter tuning, which can be time-consuming and computationally expensive. If the model is to generalize effectively across diverse medical imaging datasets, such resource demands may limit its practical use in real-world clinical environments. Recently, Song et al. introduced CenterFormer, a Transformer-based segmentation network that enhances dental plaque detection through cluster-guided attention and multi-scale feature fusion [23]. By grouping pixel features into cluster centers and integrating a Multi-Granularity Perception module, the model improves contextual understanding and boundary accuracy. It achieved state-of-the-art performance on unconstrained dental imaging datasets, demonstrating the effectiveness of advanced Transformer architectures in complex and low-contrast medical imaging scenarios.

Table 1 below provides a comparative summary of recent Transformer-based models applied in medical image segmentation. It highlights key contributions of each study, specifying the medical datasets, underlying deep learning architectures, and corresponding performance results. The models span a variety of clinical applications, from brain tumor and abdominal organ segmentation to COVID-19 lesion delineation and Alzheimer’s disease classification. Notably, the Swin Transformer and its variations (for instance, SwinUNet, SwinBTS, SUNet) have shown strong performance across multiple tasks by employing hierarchical attention mechanisms and window-based self-attention. These models consistently outperform traditional CNN-based approaches in modeling long-range dependencies while maintaining computational efficiency. The diversity of task types in the table underscores the versatility and effectiveness of ViTs in advancing medical image analysis.

## 3. Materials and Methods

In this section, we present the datasets used and describe the experimental methodology, including details of the adopted deep learning model. To mitigate the scarcity of labeled data in COVID-19 image segmentation, a large pseudo-labeled dataset was utilized during the first phase of model training. This dataset, originally introduced by Fan et al. in [8] in the context of a semi-supervised segmentation approach, provides a valuable source of auxiliary supervision that enables effective pretraining and enhances the model’s ability to generalize.

We start by providing a detailed description of the two utilized datasets, outlining the sources, characteristics, and preprocessing steps of the COVID-19 lung images for each one. Next, we discuss the model architecture, its key components, and the techniques used in developing the CoviSwin segmentation model. Finally, in the experimental setup, we elaborate on the training of the model, its hyperparameters, evaluation metrics, and the strategies employed to validate the model’s performance.

### 3.1. Dataset Description

The first COVID-19 CT segmentation dataset is known as MedSeg. It is the standard dataset employed in [24] and is composed of 100 axial CT scans from more than forty COVID-19 patients. These scans were segmented by professional doctors and radiologists based in Oslo, Norway, who were associated with the Italian Association of Medicine and Interventional Radiology. These CT scans were converted from openly accessible JPEG images to two-dimensional (2D) slices of size of 512 × 512. Each slice was annotated by a radiologist using 3 labels: ground-glass, consolidation and pleural effusion.

**Figure 1:**
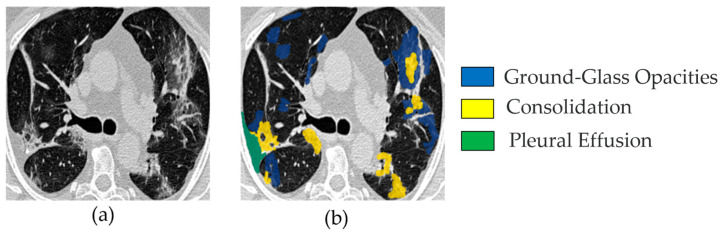
Example image from the MedSeg COVID-19 CT dataset: (**a**) one slice from a set of 100 COVID-19 CT images (named img_075.jpg); (**b**) the corresponding segmented image, showing four channels—blue for ground-glass opacities, yellow for consolidation, green for pleural effusion, and other (all remaining) areas as background.

**Figure 2:**
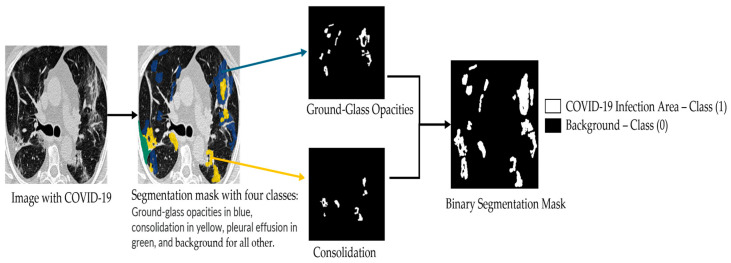
Illustration of the binary segmentation applied to each class in the multi-class segmentation map of the MedSeg COVID-19 CT slices.

Figure 1 shows a CT image and its corresponding multiclass segmentation map. In this example, four classes were annotated. To perform binary segmentation, each class was first isolated from the multiclass label and converted into a binary mask, where 0 denotes background and 1 indicates the target class, representing a COVID-19 infection area. Among the four classes, we focused on two clinically significant ones: Class 1 (ground-glass opacities, shown in blue) and Class 2 (consolidation, shown in yellow). These were merged into a single class marked in white (value = 1). The remaining two classes were grouped into the background mask (value = 0), displayed in black. This grouping step is fully illustrated in Figure 2. As a result, the segmentation is restricted to infection-specific regions while excluding unrelated anatomical structures.

We follow the same dataset split strategy as [24] for our experimental study. Specifically, the dataset is divided into training, validation, and testing subsets: 30 images are allocated for training, 20 for validation, and the remaining 50 for testing. After excluding two slices without any foreground annotations, the testing set ultimately consists of 48 images. In our work, we merge the original training and validation subsets (30 + 20 images) into a single training set, while keeping the same testing set size to ensure a fair and consistent comparison with prior studies.

**Figure 3:**
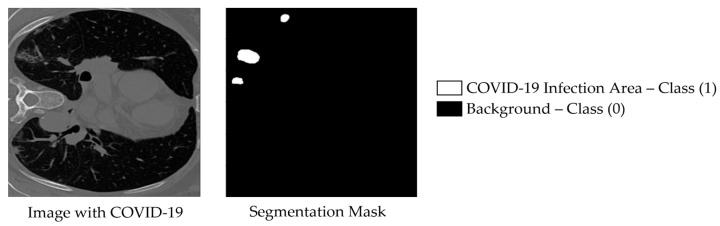
Example image from the COVID-SemiSeg dataset, named Data-1_coronacases_org_004_30, with its corresponding binary segmented ground truth mask shown on the right.

The second dataset employed in this study is the COVID-SemiSeg dataset, which was specifically curated to address the challenge of limited annotated medical images through the use of semi-supervised learning techniques. Collected by Fan et al. in the InfNet framework, the dataset contains approximately 1600 CT slices [8]. An example from COVID-SemiSeg is shown in Figure 3, depicting a COVID-19 CT scan alongside its corresponding ground-truth binary segmentation mask. In our framework, phase one leverages this pseudo-labeled dataset to pre-train the model, while the merged training set of MedSeg (50 labeled slices, originally separated into training and validation) is used as a validation set. In phase two, the merged MedSeg training set is utilized directly for fine-tuning, without maintaining a separate validation subset. This strategy is more effective than prior approaches, which trained exclusively on 30 labeled MedSeg slices, as it exploits both pseudo-labeled and fully labeled data to enhance model robustness and generalization [8].

#### Data Augmentation

In the training pipeline, we apply a limited set of data augmentation techniques to improve robustness and generalization, while avoiding excessive transformations that could unnecessarily increase model complexity. First, the input images are resized to a fixed resolution of 384 × 384, matching the required size of the SwinV2-L variant. Among the applied augmentations, geometric transformations play the most significant role: horizontal flips (with a probability of 0.5) add lateral variability, while vertical flips are less relevant in some medical cases because they may not correspond to real anatomical orientations. Random rotations within ±15 degrees and mild affine transformations, including small scaling (0.9–1.1) and translations (up to 5%), are useful because they help maintain structural integrity, yet make the model more robust to orientation and positional variations.

**Figure 4:**
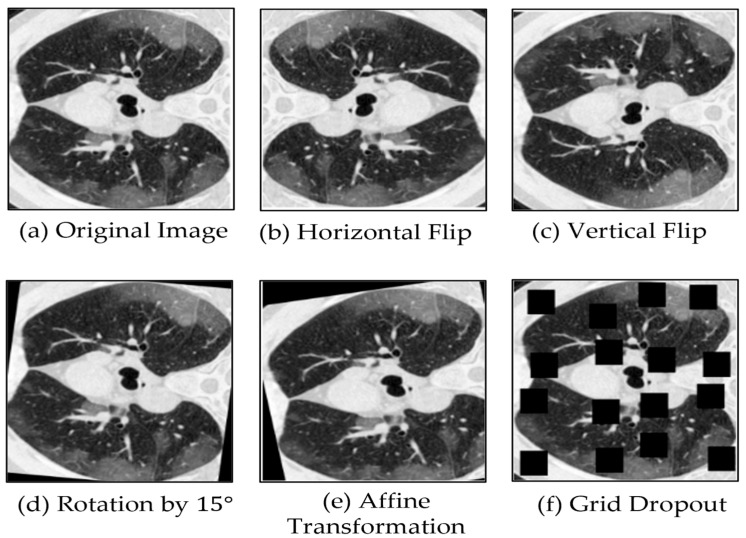
Illustration of five augmentation techniques applied to one example image. These techniques include: horizontal flip, vertical flip, rotation by 15 degrees, affine transformation, and grid dropout, respectively.

Among the key augmentation techniques employed, GridDropout plays a significant role by masking random regions of the input images, thereby introducing substantial variability into the training data. We applied a masking ratio of 0.2 (i.e., 20% of the image area). Finally, images are normalized using a mean and standard deviation of 0.5 to stabilize training, and then converted into tensors suitable for model input. Figure 4 below displays some example visualizations of the mentioned augmentation techniques.

### 3.2. Model Description

#### 3.2.1. Swin Transformer

The Swin architecture was introduced in 2021 [6]. Swin Transformer built upon the success of a Vision Transformer (ViT) architecture. However, ViT models suffer from limitations when it comes to handling a high-resolution image. In this regard, Swin transformer outperformed ViTs due to their ability to effectively handle large images with lower computational complexity. Swin Transformer is a hierarchical ViT that introduces shifted window-based self-attention to improve computational efficiency and scalability. Unlike traditional ViTs, which apply global self-attention to the entire image, Swin Transformers partition the input into non-overlapping fixed-size windows. Self-attention is computed locally within each window, significantly reducing computational complexity compared to standard ViTs. To enable information exchange between different windows, Swin Transformers incorporate a shifted window mechanism, where windows are shifted at alternating layers, allowing interactions between neighboring patches. The architecture consists of four hierarchical stages, where the feature embedding dimension doubles at each stage, similar to traditional CNNs. Each stage contains multiple Swin Transformer blocks, and each block includes: a Window Multi-head Self-Attention (W-MSA) or a Shifted-Window Multi-head Self-Attention (SW-MSA) layer, a feed-forward Multi-Layer Perceptron (MLP), Layer Normalization (LN), and Residual connections for stable optimization. The W-MSA layer operates on non-overlapping local windows whereas its SW-MSA equivalent shifts the window partition by a small offset, so that connections across multiple windows can be captured. By alternating between W-MSA and SW-MSA layers, the model effectively achieves both local and global context modeling with linear computational complexity relative to image size. Based on the window partitioning mechanism, two consecutive Swin transformer blocks can be mathematically expressed as follows:

(1) $${\hat{z}}^{l}=W-\mathrm{MSA}(\mathrm{LN}(z^{l-1}))\;+z^{l-1},$$

(2) $$z^{l}=\mathrm{MLP}(\mathrm{LN}({\hat{z}}^{l}))+{\hat{z}}^{l},$$

(3) $${\hat{z}}^{l+1}=\mathrm{SW}-\mathrm{MSA}(\mathrm{LN}(z^{l}))+z^{l},$$

(4) $$z^{l+1}=\mathrm{MLP}(\mathrm{LN}({\hat{z}}^{l+1}))+{\hat{z}}^{l+1},$$

where $z^{l}$ denotes the hidden representation features at the $l^{th}$ layer. In Equation (1), the previous layer’s output $z^{\left(l-1\right)}\;$ is normalized and processed by W-MSA, then added back via a residual connection to form ${\hat{z}}^{l}$. In Equation (2), the intermediate representation ${\hat{z}}^{l}\;$ undergoes normalization and a feed-forward MLP with a GELU (Gaussian Error Linear Unit) activation, followed by another residual addition to obtain $z^{l}$. Next, in Equation (3) a Shifted Window MSA (SW-MSA) is applied to $z^{l}$ to capture across-window dependencies, producing ${\hat{z}}^{\left(l+1\right)}$. Then, in Equation (4), a final MLP with residual connection updates the representation to ${\hat{z}}^{\left(l+1\right)}$, completing the block pair. Following the same approach described in the previous works [4,14], self-attention is computed as follows:

(5) $$\mathrm{Attention}\;(Q,\;K,\;V)\;=\mathrm{SoftMax}\left(QK^{T}/\sqrt{d}+B\right)V,$$

with Q, K, and V representing the query, key, and value matrices, respectively, while d is equal to the dimensionality of the key vectors. The term $QK^{T}$ computes the similarity between queries and keys, and dividing by $\sqrt{d}$ ensures numerical stability by preventing excessively large values. A bias term B, often used to incorporate positional information, is then added to the similarity scores. The SoftMax function converts these scores into a probability distribution that represents attention weights, determining how strongly each token attends to others. Finally, these weights are multiplied by the value matrix V to produce a weighted sum, yielding the output representation in which each token is updated as a context-aware mixture of other tokens.

**Figure 5:**
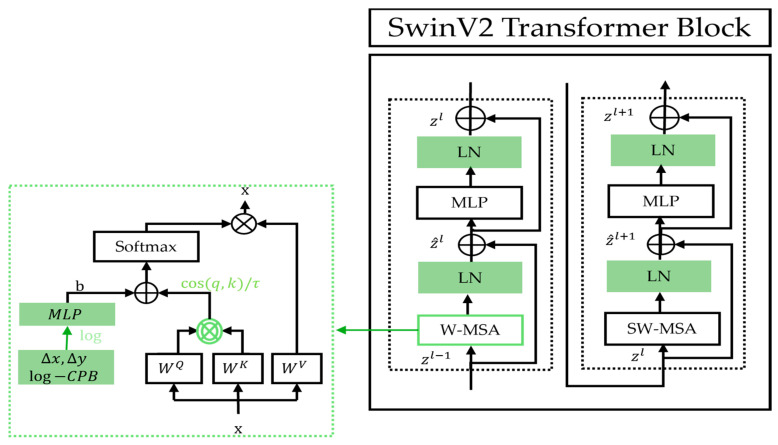
Details of SwinV2 Transformer block and its apparent modifications as compared to that of the original Swin model.

The Swin Transformer Version 2 (SwinV2) was introduced in 2022, with the objective to improve scalability to support images with extremely high resolutions as well as to overcome SwinV1’s training instability at large scales [25]. Specifically, three key modifications were proposed. The first modification involves the introduction of “res-post-norm”, which reorders the normalization layers (NL) to follow the multi-head self-attention (MSA) and multi-layer perceptron (MLP) layers, rather than preceding them. The second modification deals with the self-attention mechanism whose output incorporates a parameterized relative position bias (RPB). To address the tendency of standard attention to assign disproportionately high weights to certain input patch pairs, the authors in [25] proposed the scaled cosine attention, which normalizes the query (q) and key (k) vectors using cosine similarity before applying the outer product. Finally, the third modification, scaling up window resolution, increases the window size from 8 to 12 and raises the input resolution from 256 to 384 pixels. With fine-tuning, this improves performance. Furthermore, SwinV2 utilizes two continuous relative position bias matrices, one in linear space and one in log space, which are added to the cosine attention output. In Figure 5, we illustrate these three main modifications within the SwinV2 block.

In Table 2, we present some key characteristics of different Swin Transformer models. The number of channels in stage 1, the number of layers within each stage, and the number of model parameters show the difference(s) between the Swin Transformer types: Swin tiny (T), Swin small (S), Swin base (B), and Swin large (L). In addition, SwinV2 includes a variety of sizes, such as tiny, small, base, large, huge, and gigantic denoted by SwinV2-T, SwinV2-S, SwinV2-B, SwinV2-L, SwinV2-H, and SwinV2-G, respectively.

**Figure 6:**
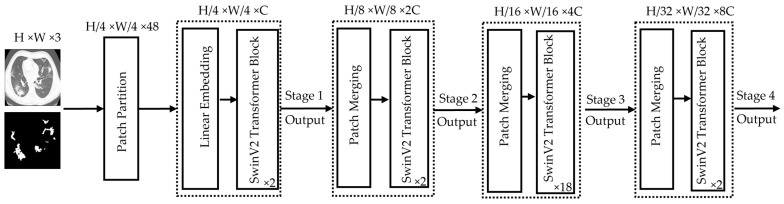
Architectural details of the encoder part used by the SwinV2-L Transformer model. The height (H) and the width (W) of the image are both equal to 384 pixels while the value 3 in H × W × 3 represents the number of channels (C) accepted by the model. The values (2, 2, 8, 2) in the bottom right-hand corner of each SwinV2 Transformer Block indicate how deep each stage is, thus referring to how many of these blocks are stacked at each resolution level.

Figure 6 illustrates the SwinV2-L Transformer architecture. The process begins with patch partitioning, where the input image is divided into smaller patches. For example, a 384 × 384 image with 4 × 4 patches results in a total of 96 × 96 = 9216 patches. A linear embedding layer then converts these patches into a sequence of tokens by embedding each patch into a vector representing its pixel values. These tokens are fed into the SwinV2-L transformer blocks, each consisting of two subunits. Each subunit includes Layer Normalization, followed by either Window-based Multi-Head Self-Attention (W-MSA) or Shifted Window Multi-Head Self-Attention (SW-MSA) (with a window size of 12 × 12 pixels), followed by another normalization layer and a Multi-Layer Perceptron (MLP). Unlike traditional self-attention, which considers all patch relationships and is computationally expensive, SwinV2-L restricts attention to local windows, improving efficiency for high-resolution images. To capture cross-window dependencies, a cyclic shift is applied before SW-MSA and reversed afterward, enabling long-range context modeling while maintaining lower computational overhead. SwinV2-L supports multiple resolutions. It was trained in this work with 384 × 384 input images. In Stage 2, SwinV2-L performs patch merging instead of processing all patches individually. Adjacent patches (typically of size 2 × 2) are concatenated along the channel dimension and reshaped into a single token, reducing spatial resolution while increasing feature dimension. The operation groups in total N × N = $N^{2}$ patches into one token. This hierarchical merging continues across stages (Stage 3, Stage 4, etc.), progressively encoding larger-scale visual patterns and capturing global context efficiently.

#### 3.2.2. CoviSwin Architecture

The proposed CoviSwin model follows a UNet-style encoder–decoder architecture optimized for high-resolution medical image segmentation. The encoder is based on SwinV2-L model, with 195.2 million parameters, pretrained on ImageNet-22K (22K classes, 14 M images), providing rich and transferable feature representations. Input images of size 384 × 384 × 3 are partitioned into non-overlapping 4 × 4 patches, reducing the spatial resolution to 96 × 96 and increasing the channel depth to 192. These patches are processed as a sequence of [9, 216, 192] tokens through four hierarchical stages of the SwinV2-L transformer. Each stage employs shifted-window multi-head self-attention (SW-MSA) and window multi-head self-attention (W-MSA) with a 12 × 12 window, progressively down-sampling spatial dimensions while increasing channel depth. The encoder produces feature maps of [96, 96, 192], [48, 48, 384], [24, 24, 768], and [12, 12, 1536], capturing increasingly abstract representations.

To enhance skip connection features for the decoder, two complementary attention mechanisms are applied to the encoder outputs: Convolutional Block Attention Module (CBAM) and Attention Gates (AGs). CBAM sequentially applies channel attention, capturing inter-channel dependencies, and spatial attention, emphasizing salient regions such as infected lung tissue. AG leverages a gating signal derived from the decoder to selectively propagate relevant encoder features [26]. Together, CBAM and AG ensure that the features passed via skip connections are task-relevant, improving segmentation accuracy, especially for heterogeneous COVID-19 lesions. The encoder also integrates Residual Convolutional Blocks (RCBs) to stabilize feature learning and enhance representational power. Each block contains two successive 3 × 3 convolutional layers, each followed by Instance Normalization and a Rectified Linear Unit (ReLU) activation, with a residual skip connection adding the input to the transformed output, formulated as:

(6) y=ReLU(x+F(x)),

where x denotes the block input and F(x) represents the learned transformation. This residual mapping preserves baseline information, mitigates the vanishing-gradient issues, and facilitates identity mappings when appropriate [27].

**Figure 7:**
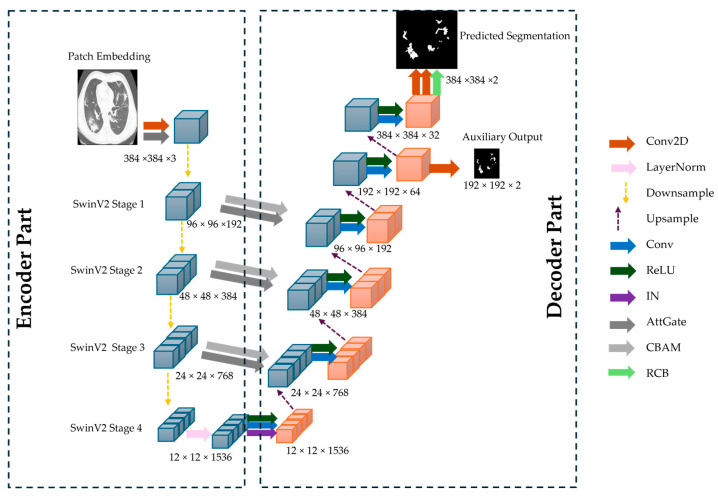
The overall architecture of the proposed CoviSwin deep learning model used in the segmentation of COVID-19 CT scans.

The decoder mirrors the encoder hierarchy and gradually reconstructs the input resolution through the use of upsampling layers. Feature maps are progressively upsampled from [12, 12, 1536] → [24, 24, 1536] → [48, 48, 1536] → [96, 96, 1536]. Skip connections from the encoder are integrated at each stage, refined using CBAM-enhanced AGs to focus on infection-specific regions. A final convolution reduces the channel depth to produce a [384, 384, 2] segmentation map, followed by a sigmoid activation. The full model architecture is shown in Figure 7.

Next, Table 3 summarizes the architectural details of the CoviSwin model, including its layer-wise parameters and feature map sizes.

CoviSwin employs a pretrained SwinV2-L encoder to capture global context via hierarchical self-attention and shifted windows, paired with a U-shaped decoder composed of upsampling blocks and convolutional refinements. We use ReLU activations and Instance Normalization (IN) within decoder blocks for the following reasons:

- ReLU provides non-saturating positive responses that maintain stronger gradients than sigmoid/tanh, improving optimization stability and convergence [28];
- IN reduces per instance covariate shift and stabilizes feature statistics across varying scanners and patients, which is advantageous in medical imaging with small to moderate batch sizes [29].

The decoder further integrates CBAM attention gates to adaptively emphasize clinically relevant structures and suppress background clutter, and residual convolutional blocks to preserve high frequency details while providing identity skip paths that facilitate gradient flow [30].

Vanishing gradients occur when gradients become extremely small as they are backpropagated through deep networks. As a result, it can stall learning in early layers, slow convergence, and yield underfit representations, especially in deep encoder–decoder and Transformer stacks [31]. Several features of our design directly mitigate this including: residual connections, which create gradient “highways” that counteract such vanishing [30]; the choice of ReLU avoids saturation regimes with near zero derivatives [32]; normalization keeps activations in a trainable range [33]; and an auxiliary output head (deep supervision) attached at an intermediate decoder stage shortens the backpropagation path and supplies strong supervisory signals to earlier layers [34]. Together, these components improve gradient propagation, training stability, and convergence, while enabling CoviSwin to capture both global context and fine anatomical detail—resulting in precise, high resolution COVID 19 lesion segmentation.

### 3.3. Experimental Setup

Our experiments were conducted using the Google Colab platform (Google LLC, Mountain View, CA, USA) with a Python version 3.12.12 Google Compute Engine backend, utilizing a T4 GPU (NVIDIA Corporation, Santa Clara, CA, USA) with 12.7 GB of system random access memory (RAM). The Adam optimizer was employed to adjust the parameters of the COVID-19 image segmentation network. To quantitatively assess the performance of the COVID-19 segmentation model, our experiments compute Recall (Sensitivity), the Dice Similarity Coefficient, and Specificity as the measures of interest.

- Recall (Sensitivity): Sensitivity evaluates the model’s ability to correctly segment COVID-19-infected lung regions. A higher sensitivity value indicates a better capability to identify and segment infected areas accurately.
- Dice Similarity Coefficient: The Dice coefficient measures the similarity between the predicted segmentation mask and the ground truth. A higher Dice score indicates better segmentation accuracy, demonstrating a stronger overlap between the predicted and actual infected regions.
- Specificity: Specificity measures the model’s effectiveness in correctly identifying non-infected lung regions. A higher specificity score signifies better segmentation performance in distinguishing healthy lung tissue from infected areas.

Higher values of these metrics correspond to better segmentation accuracy. In theory, an optimal segmentation would yield a perfect score (100%) across all metrics; however, in practice, a trade-off often arises. For instance, increasing sensitivity may lead to a decrease in specificity, and vice versa, reflecting the inherent balance between over- and under-segmentation.

The mathematical expressions for these evaluation metrics are defined as follows:

(7) $$\mathrm{Dice}\;\mathrm{Similarity}\;\mathrm{Coefficient}\;=\frac{2TP}{2TP+FP+FN},$$

(8) $$\mathrm{Recall}=\frac{TP}{TP+FN},$$

(9) $$\mathrm{Specificity}=\frac{TN}{TN+FP}.$$

In the previous three equations, True Positives (TP) denote the number of correctly identified pixels belonging to COVID-19-infected regions, whereas True Negatives (TN) indicate the number of accurately classified non-infected pixels. False Positives (FP) correspond to non-infected pixels that were incorrectly identified as infected, while False Negatives (FN) represent COVID-19-infected pixels that were mistakenly classified as non-infected.

## 4. Results and Discussion

In this section, we present the results of the proposed deep learning model, CoviSwin, for COVID-19 segmentation using CT images. Our experimental analysis includes extensive training and validation experiments, providing insights into the model’s learning behavior. We also perform a visual assessment of segmented test images to evaluate the model’s ability to accurately delineate COVID-19-infected regions. Quantitative performance is assessed using key metrics, including the Dice Similarity Coefficient, Recall, and Specificity. The following sections provide a detailed analysis of both quantitative and qualitative results obtained using the proposed approach.

### 4.1. Design of a Two-Phase Training Strategy for Segmentation Enhancement

We employ a two-phase training strategy for the segmentation of COVID-19 CT scans using the CoviSwin deep learning model, with a total of 217.16 M parameters. This training strategy is summarized in Table 4. In Phase 1, the encoder is frozen and only the decoder is trained. This yields 21.96 M trainable parameters while 195.2 M parameters stay fixed. The model was trained on the COVID-SemiSeg dataset. For 20 epochs, the training time during phase 1 took 80.76 min. We used all of the 1600 pseudo-labeled scans as a training set, while the merged set (training + validation sets) of the MedSeg dataset is used as a validation set. The model is trained for 20 epochs using a learning rate of 0.001 and a batch size of 8 due to the limitation of the GPU’s memory. The model which provided the highest DSC score on the validation set is saved as the initial model for the second phase. This phase helps the decoder learn the task and gives a strong starting point for the next phase.

In Phase 2, portions of the encoder in the last stage of SwinV2 Large model are unfrozen to allow joint training with the decoder, increasing the total number of trainable parameters to 36.16 M. Training is performed with a batch size of 8, a reduced learning rate of 0.0001, and a minimum of 50 epochs on the MedSeg dataset. Unlike previous approaches that relied on only 30 labeled images and maintained a validation split, our method utilizes all 50 labeled slices from the merged training and validation sets, without allocating a separate validation subset. Experimental results demonstrate that this strategy consistently yields superior performance compared to training on only 30 labeled images. Zhang et al. highlighted in their work that deep neural networks possess a strong capacity to fit data and generalize effectively, even without strict reliance on standard validation or regularization mechanisms [35]. This observation supports our finding that, once the model’s learning dynamics are stable, the presence or absence of a validation dataset may have minimal impact on training outcomes, especially for small datasets.

This two-phase approach helps the model transfer what it learned from the large pseudo-labeled dataset in Phase 1 to a smaller high-quality dataset, improving its general performance. This strategy uses the strengths of both datasets, large pseudo-labeled data and small high-quality labeled data, to stabilize the training and improve the performance of proposed COVID-19 CT image segmentation model.

Table 5 summarizes the improvements in segmentation performance between Phase 1 and Phase 2 and confirms the impact of this two-phase strategy. In Phase 1, the model was trained for 20 epochs using the BCE loss, achieving a Recall of 0.746, a Dice Similarity Coefficient (DSC) of 0.736, and a Specificity of 0.960. In Phase 2, after extended training for 200 epochs, the model’s performance improved to a Recall of 0.772, DSC of 0.779, and Specificity of 0.968, demonstrating enhanced sensitivity, better overlap with the ground truth, and a slightly higher ability to correctly identify negative regions.

**Figure 8:**
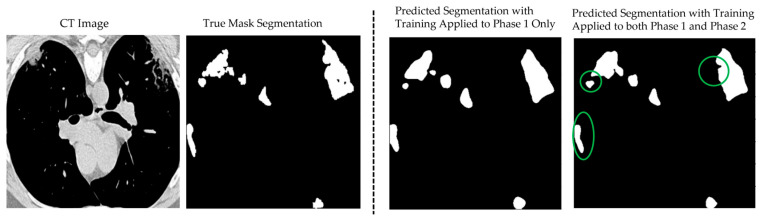
Example of the CoviSwin model prediction of COVID-19 lesion segmentation after two-phase training on image number 2 from the testing dataset in MedSeg. Clear improvements in the quality of the segmentation results can be visually noted after phase 2 compared with just phase 1, as highlighted by the shown green circles.

Figure 8 illustrates the segmentation results of the proposed CoviSwin model compared with the ground-truth mask of a sample CT scan, image number 2 from the testing dataset, after phase 1 training (third image from left) as well as after phase 2 (see the first image from the right). The latter predicted mask demonstrates a close alignment with the true lesion boundaries, capturing both the shape and extent of the affected regions with high accuracy. This improvement can be attributed to the two-phase training strategy, where the baseline model was first trained on the COVID-SemiSeg dataset and subsequently fine-tuned on the MedSeg dataset with carefully adjusted hyperparameters. As a result, the model achieved better generalization and more reliable predictions, reducing false positives and maintaining consistency with the ground-truth annotations. These results highlight measurable gains in both sensitivity and segmentation precision, while maintaining high specificity. Importantly, the improvements were achieved within a lightweight framework, reinforcing the suitability of the model for real-world deployment in resource-constrained environments such as mobile health applications and low-power diagnostic systems.

**Figure 9:**
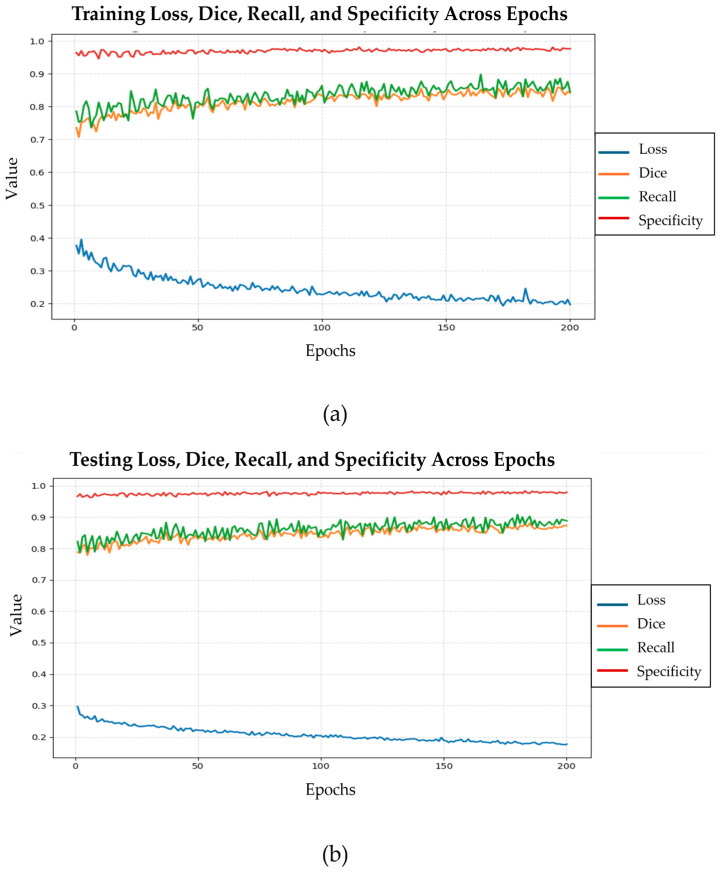
Evolution of training loss, dice similarity coefficient, recall, and specificity across 200 epochs in phase 2 using the training (**a**) and testing (**b**) datasets, respectively, for the CoviSwin model.

Figure 9 illustrates the progression of the loss, dice coefficient, recall, and specificity values across 200 epochs during phase 2, when using the training data and testing data, respectively. In part (a), the training loss decreases steadily and stabilizes after approximately 150 epochs, while both the DSC and recall scores exhibit consistent improvement, reflecting enhanced segmentation performance. Specificity remains above 0.95 throughout training, demonstrating reliable discrimination of non-target regions. These results are corroborated when the testing dataset is used, as shown in part (b) of the figure.

### 4.2. Design of a Hybrid Loss Function for Enhanced Segmentation Accuracy

Previously, the work of Panja et al. in [36] introduced the idea of a bi-category hybrid loss function, which is a composite loss that combines loss functions from two categories: one for handling pixel-wise losses to ensure the correct processing of each pixel; the other for region-boundary losses to improve the overlap and sharpness of segmentation boundaries. Likewise in this work, we propose a similar hybrid loss function that integrates three widely used loss components: Dice Loss, IoU Loss, and BCE Loss to enhance the segmentation accuracy of our model, particularly with respect to boundary alignment and regional precision. Each component contributes uniquely to addressing the common challenges in medical image segmentation, such as class imbalance, noisy annotations, and poorly defined boundaries.

- The Dice Loss is commonly used in segmentation tasks to maximize the overlap between predicted and ground truth regions. It is especially effective for handling class imbalance by emphasizing under-represented areas with fewer positive pixels. Studies have shown its superiority in segmenting regions with high class imbalance [37,38,39]. This loss function is defined as follows:
  (10) $$L_{Dice}\left(p,g\right)=1-\frac{2\sum_{i=1}^{N}{(p}_{i}g_{i})}{\sum_{i=1}^{N}\left(p_{i}\right)+\sum_{i=1}^{N}\left(g_{i}\right)+\epsilon }\;,$$
  where N is the number of pixels in each patche, p is model’s predicted probabilities per pixel i, g is the ground truth label per pixel i, and ϵ equals a small number added to avoid division by zero.

- The Intersection over Union (IoU) Loss works by maximizing the intersection and minimizing the union of the predicted and actual regions. It directly enhances spatial agreement and is particularly robust for segmenting objects with clear boundaries [40]. However, the IoU Loss can be less sensitive to smoothness, which is sometimes necessary for more gradual transitions in medical images [41,42]. This loss function is formulated in the following way:

(11) $$L_{IoU}\left(p,g\right)=1-\frac{\sum_{i=1}^{N}{(p}_{i}g_{i})}{\sum_{i=1}^{N}{(p_{i}{+g}_{i}-p}_{i}g_{i})+\epsilon }\;,$$

- The Cross-Entropy Loss is a pixel-wise classification loss that aids in fine-grained discrimination across different regions. It plays a crucial role in accurately classifying pixels, particularly in areas where boundaries are less distinct or ambiguous [41,43]. It is given by:

(12) $$L_{BCE}\left(p,g\right)=-\frac{1}{N}\;\sum_{i=1}^{N}\left(g_{i}\log\left(p_{i}\right)+\left(1-g_{i}\right)\log\left(1-p_{i}\right)\right),$$

By combining these three loss functions, obtained results from experiments using a hybrid loss function show the performance metrics of the model across different configurations, with different Gamma ($\gamma_{1},\;\gamma_{2},\;\mathrm{and}\;\gamma_{3}$) weights ranging from 0 to 1 used for each of the above three functions. The combined loss function is provided below:

(13) $$L_{combined}\left(p,g\right)=\;\gamma_{1}\times \;L_{BCE}\left(p,g\right)+\gamma_{2}\times \;L_{Dice}\left(p,g\right)\;+\;\gamma_{3}\times \;L_{IoU}\left(p,g\right),$$

Table 6 presents the Phase 1 performance of CoviSwin under different loss function configurations, trained on 1600 images from the COVID-19 SemiSeg dataset. The training was limited to 7 epochs due to time constraints, as each run required more than a week with a learning rate of 0.001 and a batch size of 8. Among the single-loss setups, Dice loss achieved the highest recall of 0.785 with a DSC value of 0.742 and specificity of 0.953. The BCE loss provided slightly lower recall at 0.770 and DSC at 0.739, while IOU loss increased recall to 0.780 but reduced DSC to 0.721, showing that it favors lesion coverage over precise boundary alignment. For dual-loss combinations, Dice + BCE improved DSC to 0.726 but reduced recall to 0.665, whereas Dice + IOU enhanced recall to 0.687 but yielded a lower DSC score of 0.717. Specificity remained high in both cases, reaching a value of 0.973 for Dice + BCE and 0.965 for Dice + IOU. The best overall balance was obtained with the three-loss hybrid function with $\gamma_{1}$ = 0.3, $\gamma_{2}$ = 0.5, $\gamma_{3}$ = 0.2, which delivered the highest recall value at 0.787 along with a strong DSC of 0.742 and an acceptable specificity of 0.944. When the weights were changed to ($\gamma_{1}$ = 0.2, $\gamma_{2}$ = 0.5, $\gamma_{3}$ = 0.3), recall decreased to 0.763 and DSC to 0.734 while specificity improved slightly to 0.951. These results confirm that IOU primarily boosts recall while lowering DSC, BCE increases DSC but tends to reduce recall, and adjusting the Gamma-weightings in the resulting hybrid losses enables a more effective balance between sensitivity, overlap accuracy, and specificity in COVID-19 lesion segmentation.

Based on these results, we employed the triple-loss configurations in Phase 2, as presented in Table 7. The model was trained for 200 epochs on the MedSeg dataset, with a reduced learning rate of 0.0001. Results show that the weightings of ($\gamma_{1}$ = 0.3, $\gamma_{2}$ = 0.5, $\gamma_{3}$ = 0.2) produced the best outcome in terms of assessment metrics, with recall = 0.817, DSC = 0.791, and specificity = 0.965. When reducing the BCE weight to 0.2, we obtained a lower performance with recall = 0.780, DSC = 0.775, and specificity = 0.964. These results demonstrate that BCE plays a crucial role in enhancing sensitivity, and that carefully tuned hybrid loss weights may allow the model to achieve a strong balance between lesion detection and segmentation accuracy across different datasets.

### 4.3. Comparisons with State-of-the-Art Methods

To evaluate the effectiveness of our proposed model, we conducted a comparison against thirteen other state-of-the-art segmentation models, including both conventional CNN-based architectures and recent Transformer-integrated models. These benchmarks span a wide range of design paradigms from encoder–decoder frameworks like U-Net [43] and U-Net++ [29] to attention-augmented networks such as Atten-Unet [44] and ACC-Unet [45], as well as Transformer-based models like TransUNet [46], Swin-Unet [7], UTNet [47], and NextSeg [48].

Each model was evaluated under identical training conditions in the work describing NextSeg [48] while using the MedSeg dataset, presented in [24], which is assessed using standard evaluation metrics such as DSC, sensitivity, and specificity. This comparison highlights the strengths and limitations of existing approaches and demonstrates the relative performance gains achieved by our model across key segmentation challenges in medical imaging. In Table 8, the presented performance comparison with various deep learning models shows that the proposed CoviSwin model performs competitively across the key evaluation metrics. The baseline model was first trained on the COVID-SemiSeg dataset for 20 epochs with a learning rate of 0.001, batch size of 8, and a triple-loss function $L_{combined}=\;{0.3\;L}_{BCE}+0.5\;L_{Dice}+0.2\;L_{IoU}$. This baseline was then further trained on the MedSeg dataset for 200 epochs with a reduced learning rate of 0.0001, while maintaining the same batch size and identical triple-loss configuration. For our proposed CoviSwin model, the generated performance values are obtained after averaging over ten experimental runs on our computing platform. The corresponding standard deviation values are also displayed. See Appendix A for more details regarding all ten runs.

From the previous table, we observe that the proposed CoviSwin model achieves average values of 0.790 ± 0.012 for sensitivity, 0.781 ± 0.0068 for the DSC, and 0.962 ± 0.0049 for specificity. In comparison, the most recent NextSeg model of 2024 achieved values of 0.731, 0.782, and 0.962, respectively. Thus, the sensitivity attained by CoviSwin is 8.07% higher than that of NextSeg, while both models yielded the same specificity and nearly identical DSC values. Notably, NextSeg’s Dice score is 0.13% higher than that of CoviSwin. The next highest sensitivity value of 0.735 was reported by the GFNet model of 2022, making CoviSwin’s sensitivity result 7.48% higher by comparison. These results suggest that the proposed CoviSwin model may offer improved segmentation of COVID-19 lesions while maintaining good performance in distinguishing non-infected regions. The observed performance can be associated with the integration of a SwinV2-L encoder, a convolution-based upsampling decoder, and Attention Gates enhanced with CBAM in the skip connections. A residual convolutional block at the output refines the predictions, whereas auxiliary and boundary heads facilitate the learning of more discriminative feature representations. Collectively, these architectural components are designed to support CoviSwin as a potentially reliable and effective framework for COVID-19 CT image segmentation.

**Figure 10:**
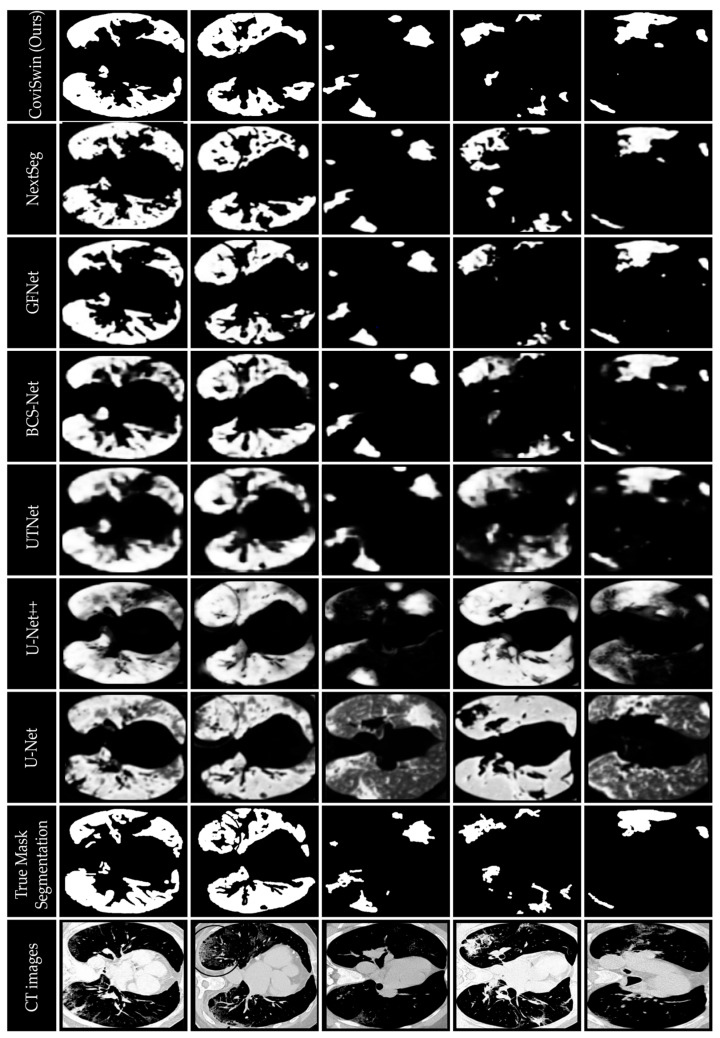
Visual comparison of segmentation results of the proposed CoviSwin model with six other state-of-the-art segmentation models showing infected regions in five selected COVID-19 CT test slices numbered 0, 1, 9, 11 and 15. The included visualization results of U-Net, U-Net++, UTNet, BCS-Net, GFNet, NextSeg were first reported in [43], [29], [47], [50], [51], and [48], respectively.

Figure 10 provides a qualitative visual comparison of lung infection segmentation results obtained from six baseline models: U-Net, U-Net++, UTNet, BCS-Net, GFNet, and NextSeg, alongside the proposed CoviSwin model, using five representative COVID-19 CT slices, as previously outlined. These five scans, taken from the test set, are numbered 0, 1, 9, 11, and 15, respectively. The comparison shows that the segmentation masks generated by CoviSwin appear to align more closely with the radiologist-annotated ground truth. In particular, the model tends to capture both consolidation and ground-glass opacity regions with improved completeness and fewer apparent false-positive activations relative to the other models. These observations suggest that CoviSwin provides more consistent and reliable delineations of infection regions compared with existing approaches.

## 5. Conclusions

In this article, we propose CoviSwin, a U-shaped encoder–decoder deep learning network that leverages a Transformer-based architecture for high-precision medical image segmentation. The model combines a SwinV2-L encoder, CBAM-enhanced Attention Gates, and Residual Convolutional Blocks, enabling effective extraction of both global contextual information and fine-grained spatial details from 2D COVID-19 CT images. CoviSwin attains ten-run averages of 0.790 ± 0.012 for sensitivity, 0.781 ± 0.0068 for DSC, and 0.962 ± 0.0049 for specificity based on our experimental evaluation. Relative to the obtained results of NextSeg in 2024, where a sensitivity of 0.731, a DSC of 0.782, and a specificity of 0.962 are reported, our model attains a higher average sensitivity under our experimental setup while maintaining a comparable specificity and a broadly similar DSC. Overall, CoviSwin’s performance indicates a strong ability to segment COVID-19-infected regions while reliably distinguishing non-infected areas. We attribute this performance to the integration of Transformer-based feature extraction, attention mechanisms, and residual connections, which together may contribute to a more robust framework for medical image segmentation.

Regarding clinical deployment, inference speed is generally not a limitation—modern deep learning models achieve near real-time performance when deployed on GPU-enabled platforms. The primary challenge lies in maintaining high segmentation accuracy when the model is introduced to new clinical environments. This difficulty, widely recognized in deep learning applications, arises from domain shifts and data distribution variability, which often degrade model performance over time and necessitate retraining. Moreover, deep learning models typically require substantial amounts of annotated data, which is not always feasible in clinical settings.

Our approach mitigates these challenges by pretraining the model on a large pseudo-labeled COVID-19 dataset, establishing strong feature representations that generalize well across domains. When deployed in a new clinical site, only the second training phase is required—fine-tuning the pretrained model on a small number of locally labeled samples (e.g., 50 images as demonstrated). This process is computationally efficient and enables the model to achieve comparable performance to the original results.

Future work will focus on further improving generalization across diverse datasets, exploring domain adaptation strategies, and optimizing computational efficiency for seamless integration into clinical workflows.

## Figures and Tables

**Table 1:** Summary of some recent works using Transformer-based models and their performance results on medical imaging datasets.

Year, Ref.	Deep Learning Model	Description	Reported Results
2022, [14]	Class-Aware Adversarial Transformer (CATformer, and CASTformer)	Transformer segmentation models (CATformer and CASTformer) of medical structures, such as tumors or organs.	Average Dice = 82.17 and 82.55
2022, [20]	SwinBTS	3D Swin Transformer model for brain tumor segmentation using multi-modal MRI datasets (BraTS).	Average Dice = 81.15
2022, [21]	Swin Transformer	Swin Transformer model for multi-organ segmentation (AMOS) of abdominal CT images.	Mean Dice = 84.91 (in-distribution) and 87.20 (out-of-distribution)
2023, [18]	ARSeg, MedT, TransUNet, TransM, and UNeXt	ViTs for lung segmentation of COVID-19 using CXR images.	F1 Score = 95.36, 97.40, 96.80, 97.47, and 96.26
2023, [15]	Swin Transformer with CNN	CNNs and Swin Transformer combined to classify Alzheimer’s Disease Neuroimaging Initiative (ADNI) data using brain imaging.	Accuracy = 82.09% Sensitivity = 86.96%
2024, [22]	Transformers with Learnable Token Merging (LTM)	ViT-based models using learnably merging semantically similar tokens on ImageNet-1K benchmark.	Top-1 Accuracy = 83.64% (LTM-Swin-B after 50 epochs)
2024, [23]	CenterFormer Model	Cluster center enhanced Transformer for dental plaque segmentation on the dental plaque dataset.	Pixel Accuracy = 76.81%

**Table 2:** Listing of different Swin Transformer models and their characteristics. Those of the SwinV2-L model are displayed in bold to highlight their importance to our CoviSwin model.

Type of Swin Transformers	Number of Channels (Stage 1)	Number of Layers per Stage	Number of Parameters (in Millions)	Image Resolutions
Swin-T	96	(2,2,6,2)	28	224 × 224
Swin-S	96	(2,2,18,2)	50	224 × 224
Swin-B	128	(2,2,18,2)	88	224 × 224/384 × 384
Swin-L	192	(2,2,18,2)	197	224 × 224/384 × 384
SwinV2-T/S/B	96/96/128	(2,2,6,2)	28/50/88	256 × 256
**SwinV2-L**	**192**	**(2,2,18,2)**	**197**	256 × 256/384 × 384
SwinV2-H	352	(2,2,18,2)	658	256 × 256/512 × 512
SwinV2-G	512	(2,2,42,4)	3000	1024 × 1024

**Table 3:** CoviSwin model’s architecture parts with parameter details and layer specifications.

Parts	Layers		Input Shape	Output Shape
Encoder	Patch Embedding	Conv2D	[1, 3, 384, 384]	[1, 192, 96, 96]
		LayerNorm	[1, 192, 96, 96]	[1, 192, 96, 96]
	SwinV2_Stage1		[1, 192, 96, 96]	[1, 384, 48, 48]
	SwinV2_Stage2		[1, 384, 48, 48]	[1, 768, 24, 24]
	SwinV2_Stage3		[1, 768, 24, 24]	[1, 1536, 12, 12]
	SwinV2_Stage4	LayerNorm	[1, 1536, 12, 12]	[1, 1536, 12, 12]
Decoder	UpsampleAttnBlock3	Upsample + Conv + ReLU + IN	[1, 1536, 12, 12]	[1, 768, 24, 24]
		AttentionGate_3 + CBAM	[1, 768, 24, 24]	[1, 768, 24, 24]
	UpsampleAttnBlock2	Upsample + Conv + ReLU + IN	[1, 768, 24, 24]	[1, 384, 48, 48]
		AttentionGate_2 + CBAM	[1, 384, 48, 48]	[1, 384, 48, 48]
	UpsampleAttnBlock1	Upsample + Conv + ReLU + IN	[1, 384, 48, 48]	[1, 192, 96, 96]
		AttentionGate_1 + CBAM	[1, 192, 96, 96]	[1, 192, 96, 96]
	UpsampleBlock0	Upsample + Conv + ReLU + IN	[1, 192, 96, 96]	[1, 64, 192, 192]
	UpsampleBlock192to384_	Upsample + Conv + ReLU + IN	[1, 64, 192, 192]	[1, 32, 384, 384]
	Auxiliary Head	Conv2D	[1, 64, 192, 192]	[1, 2, 192, 192]
	Boundary Head	Conv2D	[1, 32, 384, 384]	[1, 2, 384, 384]
	Output Conv	Conv2D + ResidualBlock	[1, 32, 384, 384]	[1, 2, 384, 384]

**Table 4:** Trainable and frozen parameters in each phase of using CoviSwin model, highlighting the decoder and encoder updates and the total number of model parameters. The latter is given in millions.

Phase	Trainable Parameters	Frozen Parameters	Training Time (20 Epochs)	Notes
Phase 1 (Frozen Encoder)	21.96 M	195.2 M	80.76 min (1.35 h)	Only Decoder is Updated
Phase 2 (Partially Unfrozen Encoder)	36.16 M	181.0 M	11.53 min (0.19 h)	Decoder + Part of the Encoder are Updated

**Table 5:** Comparison of segmentation performance metrics between Phase 1 and Phase 2. Higher values are depicted in bold.

Phase	Recall	DSC	Specificity
Phase 1	0.746	0.736	0.960
Phase 2	**0.772**	**0.779**	**0.968**

**Table 6:** Performance comparison of the proposed CoviSwin model when using different loss function configurations on the testing dataset. Higher values are shown in bold.

Phase	Loss Function	Recall	DSC	Specificity
Phase 1	$L_{Dice}$	0.785	**0.742**	0.953
	$L_{BCE}$	0.77	0.739	0.95
	$L_{IoU}$	0.78	0.721	0.95
	$L_{combined}={0.5\;L}_{BCE}+0.5\;L_{Dice}$	0.665	0.726	**0.973**
	$L_{combined}=0.5\;L_{Dice}+0.5\;L_{IoU}$	0.687	0.717	0.965
	$L_{combined}={0.3\;L}_{BCE}+0.5\;L_{Dice}+0.2\;L_{IoU}$	**0.787**	**0.742**	0.944
	$L_{combined}={0.2\;L}_{BCE}+0.5\;L_{Dice}+0.3\;L_{IoU}$	0.763	0.734	0.951

**Table 7:** Segmentation performance results of CoviSwin model across selected loss function configurations used in Phase 2 on the testing dataset. Higher values are exhibited in bold.

Phase	Loss Function	Recall	DSC	Specificity
Phase 2	$L_{combined}={0.3\;L}_{BCE}+0.5\;L_{Dice}+0.2\;L_{IoU}$	**0.817**	**0.791**	**0.965**
	$L_{combined}={0.2\;L}_{BCE}+0.5\;L_{Dice}+0.3\;L_{IoU}$	0.78	0.775	0.964

**Table 8:** Segmentation performance comparison of CoviSwin model, over ten experimental runs showing the mean and standard deviation (SD) values, with thirteen other State-of-the-Art segmentation models on the testing dataset. Highest values are shown in bold while the next-to-highest ones are underscored.

Model [Ref.], Year	Sensitivity	DSC	Specificity
U-Net [18], 2023	0.653	0.632	0.917
U-Net++ [29], 2018	0.638	0.623	0.935
Inf-Net [8], 2020	0.692	0.682	0.944
Semi-Inf-Net [8], 2020	0.725	0.739	0.96
MSNet [49], 2020	0.718	0.773	0.954
Atten-Unet [44], 2018	0.673	0.65	0.924
UTNet [47], 2021	0.694	0.695	0.932
TransUnet [46], 2021	0.722	0.712	0.953
BCS-Net [50], 2022	0.709	0.763	0.946
Swin-Unet [7], 2021	0.713	0.689	0.938
GFNet [51], 2022	0.735	0.754	0.957
ACC-Unet [45], 2023	0.724	0.768	0.949
NextSeg [48], 2024	0.731	**0.782**	**0.962**
**CoviSwin (Ours), 2025** **Mean ± SD**	**0.790 ± 0.0120**	0.781 ± 0.0068	**0.962 ± 0.0049**

## Data Availability

Dataset MedSeg used in the study can be downloaded from https://www.kaggle.com/competitions/covid-segmentation/data?select=images_medseg.npy, last accessed: 10 June 2025. Dataset COVID_19 SemiSeg used in the study can be downloaded from https://github.com/DengPingFan/Inf-Net?tab=readme-ov-file, last accessed: 12 June 2025.

## Abbreviations

The following abbreviations are used in this manuscript:

AMOS	Abdominal Multi-Organ Segmentation
ADNI	Alzheimer’s Disease Neuroimaging Initiative
AG	Attention Gate
BCE	Binary Cross-Entropy
CXR	Chest X-Ray
CGS	Collaborative Generalist and Specialists
CT	Computed Tomography
CBAM	Convolutional Block Attention Module
CNNs	Convolutional Neural Networks
COVID-19	“CO” stands for corona, “VI” stands for virus, “D” stands for disease, and “19” stands for 2019
DSC	Dice Similarity Coefficient
FN	False Negative
FP	False Positive
GELU	Gaussian Error Linear Unit
GPU	Graphics Processing Unit
HVTs	Hybrid Vision Transformers
IN	Instance Normalization
IoU	Intersection over Union
KiTS	Kidner Tumor Segmentation
LN	Layer Normalization
LTM	Learnable Token Merging
MRI	Magnetic Resonance Imaging
MTAN	Mean Teacher Attention N-Net
MSA	Multi-head Self-Attention
MLP	Multi-Layer Perceptron
NL	Normalization Layer
PSNR	Peak Signal-to-Noise Ratio
RAM	Random Access Memory
ReLU	Rectified Linear Unit
RPB	Relative Position Bias
RCB	Residual Convolutional Block
SARS	Severe Acute Respiratory Syndrome
SARS-CoV-2	Severe Acute Respiratory Syndrome CoronaVirus 2
SD	Standard Deviation
SSIM	Structural Similarity Index
Swin	Shifted Window Transformer
SwinV2	Shifted Window Transformer Version 2
SW-MSA	Shifted Window Multi-Head Self-Attention
TN	True Negative
TP	True Positive
US	UltraSound
ViTs	Vision Transformers
W-MSA	Window-based Multi-Head Self-Attention
WHO	World Health Organization

## Appendix A

In this appendix, we provide the performance results of our CoviSwin model for the segmentation of COVID-19 CT scans over ten runs. We report therein the mean and standard deviation values as well.

**Table A1:** Performance results of ten runs of our proposed Transformer model, CoviSwin, used for the segmentation of COVID-19 CT scans. The mean and standard deviation values are also included. Highest values are shown in boldface while means are underlined.

Run Number	Sensitivity (Recall)	Dice Similarity Coefficient (DSC)	Specificity
1	0.797	0.773	0.955
2	0.787	0.782	0.961
3	0.772	0.779	0.968
4	0.786	**0.791**	**0.969**
5	0.783	0.781	0.963
6	0.782	0.782	0.964
7	0.792	0.782	0.962
8	0.797	0.773	0.955
9	0.789	0.772	0.958
10	**0.817**	**0.791**	0.965
Mean	0.790	0.781	0.962
Standard Deviation (SD)	0.011988884	0.006785606	0.004876246
